# Navigating Without a Compass: A Qualitative Study on Parents' Infant Formula Handling

**DOI:** 10.1002/fsn3.71432

**Published:** 2026-01-07

**Authors:** Erika Andresen, Paola Oras, Gunilla Norrman, Mats Målqvist, Eva‐Lotta Funkquist

**Affiliations:** ^1^ Department of Women's and Children's Health Uppsala University Uppsala Sweden; ^2^ Centre for Research and Development Uppsala University, Region Gävleborg Gävle Sweden; ^3^ The Paediatric Clinic, Hudiksvall Hospital, Region Gävleborg Hudiksvall Sweden

**Keywords:** hygiene, infant, infant formula, parents, powdered infant formula, qualitative research, safety

## Abstract

The WHO guidelines on safe formula feeding emphasize the importance of proper handling with professional guidance. However, many infants worldwide are given unsafe formula, with parents struggling with handling procedures. Despite the prevalence of formula feeding, research on formula handling in Swedish settings is limited. This study explores and describes parents' practices and experiences in handling infant formula, their experiences of information on safe handling and their potential needs. Individual semi‐structured interviews were conducted with 15 mothers who had experienced formula feeding their infants. Data were transcribed verbatim and analyzed using qualitative content analysis. One overarching theme was identified: “Navigating alone without a compass, with a desire for a safe journey”, along with four categories: (1) Varying degrees of safe handling, (2) Challenging but doable, (3) The centrality of guidance and support, and (4) Aspects that also matter. Mothers used varying degrees of safe methods for preparation, storage and cleaning. Although they perceived the methods as feasible, they encountered numerous challenges. Moreover, they received insufficient professional information, relying mainly on informal sources. While guidance and support were requested, they also desired inclusion, as the lack of information and support caused feelings of shame and guilt. Positive emotions surrounding formula availability, shared feeding responsibilities and increased parental freedom influenced its use and handling, as did cost‐related constraints. This study suggests that guidance on safe formula feeding is not a priority in the healthcare continuum. To promote optimal health in infants, the results highlight the need to review the quality, implementation, understanding, and adherence to national and local formula feeding guidelines.

## Graphical abstract

Proper handling of infant formula, along with professional guidance, is essential to prevent health risks among formula‐fed infants in both high‐ and low‐income countries. Results reveal that parents use unsafe preparation, storage and cleaning methods when handling infant formula, find these practices challenging, and lack adequate information and support. To promote optimal health in infants, results suggest a need to review national and local guidelines in relation to the WHO guidelines on the safe handling of infant formula, assess their implementation, and ensure they are understood and followed within the healthcare system without interfering with breastfeeding.

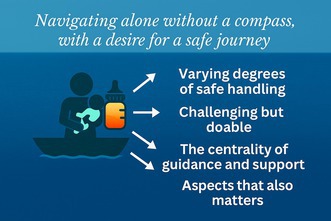

## 1 Introduction

Each child has the right to the highest attainable standard of health, for which a safe feeding method is essential (UNHCR 2016). The World Health Organization (WHO) recommends exclusive breastfeeding the first 6 months, followed by partial breastfeeding up to 2 years or longer, together with safe complementary foods (WHO and UNICEF 2018). Nevertheless, less than 50% of infants are exclusively breastfed at 6 months (UNICEF 2023), and the use of commercial infant formula is widespread (Neves et al. 2021). In Sweden, between 13% and 40% of full‐term, normal‐sized, newborns receive infant formula in hospital after birth (Skogdal et al. 2025), and fewer than 12% of infants are exclusively breastfed at 6 months (National Board of Health and Welfare 2024).

Unsafe feeding practices, including improper handling and contamination of infant formula, have been reported for decades in both high‐ and low‐income countries (Andresen et al. 2007; EFSA and ECDC 2018; Ellison et al. 2017; Henry and Fouladkhah 2019; Leung et al. 2009; Renfrew 2003; Rothstein et al. 2019; Surjono et al. 1980), posing a serious risk of illness and death in infants (Agostoni et al. 2004; Henry and Fouladkhah 2019; Strysko et al. 2020).

To reduce the risk of illness and death in infants from contaminated powdered infant formula (PIF), the WHO issued safety guidelines in 2007 (WHO 2007) for both high‐ and low‐income countries. These guidelines focus on the safe preparation and storage of infant formula and hygiene, cleaning, and sterilization of feeding equipment. Despite these guidelines, research shows they are often not followed. Commonly reported issues include incorrect formula concentration, the addition of cereals (Appleton et al. 2022; Lakshman et al. 2009), poor hand hygiene (Carletti and Cattaneo 2008; Grant et al. 2024; Labiner‐Wolfe et al. 2008), and misunderstandings about bacterial contamination risks (Calamusa et al. 2009; Grant et al. 2024; Labiner‐Wolfe et al. 2008). Additionally, parents often do not read or fully understand instructions and store prepared formula longer than recommended (Grant et al. 2024; Labiner‐Wolfe et al. 2008). Guidance on infant feeding is perceived as ineffective (Lagan et al. 2014), with limited information on formula feeding (Appleton et al. 2018; Hvatum and Glavin 2017; Lagan et al. 2014). Furthermore, parents often rely on informal sources (Appleton et al. 2020) and start giving infant formula before receiving advice from health professionals (Appleton et al. 2022).

Healthcare professionals in Sweden are expected to provide information and guidance on infant feeding to caregivers. Where there is a need for infant formula, this includes proper use of formula and health risks associated with improper use (National Board of Health and Welfare 2017). The WHO safety guidelines recommend mixing PIF with boiled water at a temperature of no less than 70°C to eliminate potentially harmful pathogens. Guidelines highlight that each feed should be freshly prepared, rapidly cooled, and served immediately. Leftovers should be discarded if not consumed within 2 h. They also emphasize the importance of an accurate water‐to‐powder ratio and advise against microwave heating. Where available, WHO recommends using sterile ready‐to‐feed formula for infants at greatest risk (WHO 2007). In contrast, Swedish healthcare guides differ in several respects. They recommend using good‐quality tap water and preparing formula according to the manufacturer's instructions. Sterilizing feeding utensils after use is considered unnecessary if they have been thoroughly washed with hot water, dishwashing liquid, and a dish brush. Additionally, leftovers are advised to be discarded after 1 h, and no guidance is provided regarding ready‐to‐feed formula for infants at greatest risk (1177 2022; The National Handbook for Child Health Services 2022).

A Swedish study on introducing solid foods found that parents often experience stress, uncertainty, and feel responsible for doing it ‘right’, highlighting the need for more information and support (Henström et al. 2020). Little is known about how parents handle and experience infant formula in Sweden. Moreover, there is limited knowledge about the information parents receive regarding safe infant formula handling, how they perceive this information and what additional support they feel they need. This study aimed to explore and describe parents' practices and experiences with handling infant formula, their experiences of information on safe handling and their potential needs.

## 2 Methods

### 2.1 Design and Study Setting

The study had a qualitative, exploratory, descriptive design as part of a Swedish research project on evaluating and developing a breastfeeding support program (Oras 2020). The Swedish Ethical Review Board approved the study (Dnr 2020‐01417), which was conducted in one of Sweden's central regions. To enhance transparency, the Standards for Reporting Qualitative Research were followed (O'Brien et al. 2014).

### 2.2 Participants and Recruitment Process

Eligible participants were parents aged 18 or older living in Sweden, Swedish speaking, with an infant under 12 months, and experience providing infant formula. A purposive sampling method was used to select information‐rich, illuminative interviewees (Patton 2015). Recruitment occurred through nurses or midwives at child and maternal healthcare centers, or by the first author (E.A.) at open preschools (where infants attended with their parents) in the same buildings. Oral and written information was distributed, and interested individuals provided their contact details for SMS follow‐up by E.A., who arranged interviews shortly after. Participants chose between physical meetings, phone interviews, or Zoom. If there was no response to the initial SMS, parents were contacted once more via SMS and email. The final number of interviewees was determined by the research team based on data richness, with a focus on content variation and multiplicity (Graneheim et al. 2017). In total, 17 parents provided their contact details, of which two did not respond. Eight interviews were conducted via telephone, five in person, and two via Zoom.

### 2.3 Data Collection

Individual interviews were conducted between March 2023 and January 2024 by E.A., who had no prior relationship with the participants. Oral and written consent were obtained, and all interviews were conducted in Swedish. Semi‐structured interviews explored parents' practices and experiences. An interview guide, developed by E.A., included background questions and topic‐specific questions based on the WHO's guidelines for the safe handling of infant formula (WHO 2007). Questions around the information were added. Probes were used to expand or clarify responses (Robinson 2023). At the end of each interview, participants were asked if there was anything they wished to add regarding infant formula that had not been covered. The interview guide was reviewed by the research team, all of whom had expertise in infant feeding and experience in qualitative research. Two pilot tests were performed, and minor language adjustments were made to the interview guide; however, the pilot interviews were not included in the study. Interviews included in the study lasted between 22 and 47 min, were audio recorded, and transcribed verbatim in Swedish by E.A.

### 2.4 Data Analysis

Data were analyzed using qualitative content analysis (Graneheim and Lundman 2004), with an inductive approach to identify patterns (Graneheim et al. 2017). This multi‐step method balances between descriptions of manifest content and interpretations of latent content, while staying close to participants' experiences. Initially, E.A. read transcripts multiple times to gain an overall sense of the data. Meaning units related to the aim were identified, extracted, and condensed. Condensed meaning units were labeled with descriptive codes, closely aligned with the text. Codes with similar content were sorted, interpreted, and abstracted into sub‐categories, which were then compared and grouped into categories describing the manifest content. The research team discussed the sorted codes and the underlying meanings of the sub‐categories and categories in a reflexive manner, interpreting and abstracting them into a theme that describes the latent content. The analysis was iterative, with codes, sub‐categories, categories, and themes compared and discussed; see Table 1. Adjustments were made until a consensus was reached among all authors. The sub‐categories, categories, and theme were translated into English by E.A. upon identification. Quotes were translated into English by a professional translator.

**TABLE 1:** Example of the analysis process.

Condensed meaning unit	Code	Sub‐categories	Categories	Theme
Now that she is a little older, we have switched to giving cold water from the tap. We take cold water from the tap in the bottle and then the same procedure that we heat in the microwave and mix with right number (scoops)	Nowadays, cold water from the tap, heat in microwave and mix with right number (scoops)	Preparation variations including adaptations and exceptions	Varying degrees of safe handling	Navigating alone without a compass, with a desire for a safe journey
If you're in a place and you can't get enough hot water in the tap, then it feels a bit disgusting, unhygienic when you can't get rid of this film of fat	Sometimes difficult to remove coating if not enough hot water, feels disgusting, unhygienic	Positive and negative cleaning and hygiene experiences	Challenging but doable	
I feel like I've covered everything to see what it says most of	Read everywhere to compare	Guided through own choices and informal sources	The centrality of guidance and support	

## 3 Results

In total, 15 interviews were conducted with caregivers living in different areas of the region. The participants were females who identified themselves as mothers. Table 2 presents the background characteristics for both mothers and infants, and the use of infant formula at the time of the interview.

**TABLE 2:** Participants' characteristics (*n* = 15).

Age (year)	25–38
Country of birth	
Sweden	13
Other	2
Level of education	
High school	4
Vocational program	2
University/college	9
Civil status	
Partner	12
No partner	3
Number of children	
One	10
Two	4
Three	1
Earlier experience of infant formula	
Yes, own child	5
Yes, other's child/children	4
No	6
Age of infant	
< 2 months	0
2–3 months	4
> 3–5 months	5
6–7	1
8–12	5
When infant formula was first given	
Within the first hours	3
Within the first days/week	6
Within the first month	3
Between 1–4 months	3
Infant still receiving infant formula	
Yes fully	8
Yes partly	6
No	1
Type of formula used	
PIF	10
Ready‐to‐feed	1
Both	4
Feeding by bottle or cup	
Bottle	14
Both	1
No. of bottles	2–several
Infant formula machine	4

### 3.1 Navigating Alone Without a Compass, With a Desire for a Safe Journey

The theme “Navigating alone without a compass, with a desire for a safe journey,” derives from four categories, each representing an aspect of the theme. These categories are: (1) Varying degrees of safe handling, (2) Challenging but doable, (3) The centrality of guidance and support, and (4) Aspects that also matter. The first category relates to parents' practices in handling infant formula, the second to their experiences of the handling process, the third to their experience of information, and the fourth to experiences of influencing aspects. The categories are further divided into 14 sub‐categories, which are summarized in Table 3, together with illustrative quotations.

**TABLE 3:** Results of the data analysis.

Theme	Categories	Sub‐categories
Navigating alone without a compass, with a desire for a safe journey	Varying degrees of safe handling	Preparation variations, including adaptations and exceptions
		Storage variants of infant formula and equipment
		Mainly freshly made formula feeds and discarding of leftovers
		More or less thorough cleaning and hygiene
	Challenging but doable	Stress and challenges before manageable preparation
		Needs for own solutions for impractical packaging and storage
		Positive and negative cleaning and hygiene experiences
		Safe feeds and cleanliness important, but perceptions and adaptations differ
	The centrality of guidance and support	Absent or insufficient formal information
		Guided through own choices and informal sources
		The importance of guidance
		A taboo subject or a good option? The desire for acceptance and inclusion
	Aspects that also matter	The importance of feeling secure and free
		Suitability and preferences matter, but cost can be a barrier

### 3.2 Category 1: Varying Degrees of Safe Handling

#### 3.2.1 Preparation Variations, Including Adaptations and Exceptions

Preparation practices for infant formula varied, including adaptations and exceptions. It was prepared by mixing the PIF with pre‐warmed tap water, precooked cooled water, or cold tap water. Precooked water could be stored hot or warm in a thermos or cooled in a jug or glass bottle. If cold water was used, it was commonly heated to feeding temperature using a microwave. Keeping the bottle under running hot tap water or using a bottle warmer was also mentioned. Some mothers reported taking more care to boil the water when their infant was younger, and some mothers stated that they sometimes served the formula cold or at room temperature. No one reported mixing the powder with hot water. The correct amount of water was typically poured into the bottle before the powder was added, except in one case where a mother mentioned doing the opposite. This mother also reported adding small amounts of cream, extra powder, or water while mixing the formula.

The powder and then the water… shake. Sometimes I added some cream because they [children] were so small… It has happened sometimes [extra powder]… maybe it helps them get a healthier body… then I think ‘no, maybe you get too much’… then I go back and add some more water. But I've always tasted… If I think it's good, then they think so too… (R10)

Mothers described using leveled scoops, either by dragging the scoop along the edge or by using a leveling tool. Those using ready‐to‐feed formula heated it in the microwave, kept the bottle under running warm tap water, or served it cold. Those using formula machines filled the machine with tap water or pre‐boiled water, receiving formula that was ready to drink directly.

#### 3.2.2 Storage Variants of Infant Formula and Equipment

Some mothers stored PIF in its original packages, while several transferred the powder to plastic or glass jars, including dosing containers. Those using formula machines filled the cistern with powder and kept any residues in the original packaging or glass jars. The formula was stored in the pantry, on designated kitchen counter space, or in a room set aside for infant formula‐related items. Opened ready‐to‐feed formula was stored in the fridge or in a cooler if away from home.

We have stored the powder in another plastic container. At night, when we didn't use ready‐to‐feed [formula], we had these little powder dispensers. (R12)

Some mothers stored cleaned feeding equipment in dedicated places on the counter, mounted in separate cupboards, or with other items. Others stored it on clean towels, drying mats, or a separate dish or bottle rack. Measuring spoons were either stored inside the jar or attached to the outside.

#### 3.2.3 Mainly Freshly Made Formula Feeds and Discarding of Leftovers

Most mothers prepared fresh formula for each meal, serving it immediately or within 1 h. Leftovers were usually discarded after 1 h, and sometimes within 15–30 min. One mother mentioned saving formula for a few hours before reheating it, while another allowed the baby to drink from the same feed for several hours and used a bottle warmer for leftovers.

I used [bottle warmer] until he was six months old. But now, when he is older…I give it for many hours… when five hours have passed… or four… I just change. (R10)

#### 3.2.4 More or Less Thorough Cleaning and Hygiene

Some mothers described a cleaning process involving rinsing, washing in hot water with dish soap and a bottle brush, and then rinsing again, while several others used simpler methods. Water temperatures ranged from hot to lukewarm. Most mothers stated they always used washing‐up liquid, while a few rarely or never did. Several used bottle brushes, some used separate dish brushes, and others used regular brushes. One mother used a dish sponge, and some used their hands instead of a brush. A few occasionally used the dishwasher. None of them sterilized the equipment after each use; sterilization ranged from daily to every 2 weeks, or never. For separable bottles, whether they were unscrewed after each use for cleaning varied.

Either I go and rinse them out immediately, so that they are rinsed out anyway, or I usually use a new one each time. (R13)

One mother, using an infant formula machine, regularly cleaned the hose after every fourth use, as recommended, and the water cistern when changing the water. The powder compartment had been rinsed once in 4 months. Another mother had cleaned the hose more frequently when the child was younger but cleaned all other parts of the machine once a month, per recommendations.

Washing hands with soap before preparing formula was described as occurring more often or sometimes, rather than always. A few mothers always did it, while several mentioned they had done it more often when the infant was younger.

I don't know if it is specifically before formula, but it's very, very often anyway per day. (R14)

### 3.3 Challenging but Doable

#### 3.3.1 Stress and Challenges Before Manageable Preparation

Most mothers described preparing formula as manageable, though several found it stressful, difficult, and messy. There were concerns about not mixing it correctly. Practical challenges included simultaneously holding a crying baby, ensuring the right amount of water and powder, achieving the correct feeding temperature and quantity, or mixing formula outside the home.

…when you are very, very tired and then she just screams, and then it happens… that you lose count… It has happened once or twice that it became completely crazy. (R11)

Some expressed that breastfeeding would have been easier compared to preparing formula. Measures that made preparation easier included pre‐boiling water, storing chilled water in the fridge and hot water in a thermos, preparing portions of powder in advance, and anticipating the child's needs. Using ready‐to‐feed formula or a formula machine was also appreciated.

#### 3.3.2 Need for Own Solutions for Impractical Packaging and Storage

Several mothers found the original packaging of PIF impractical and cramped, leading to create their own storage solutions, while others using different types did not.

It was tricky to get… because you had hands that were too big to reach down, and be able to get the right amount in the scoop…. then we felt it was easier to pour it over. (R15)

Ready‐to‐feed formula was considered impractical and restrictive once opened, as it had to be kept cold and consumed within a certain timeframe.

#### 3.3.3 Positive and Negative Cleaning and Hygiene Experiences

Most mothers found cleaning manageable, though some found it difficult, stressful, or unappreciated. Bottle brushes, regular dish brushes, and separable feeding bottles were described as helpful, along with filling bottles and teats with water to prevent residue build‐up if cleaning was delayed. One mother, who rarely used dish soap or a dish brush, sometimes had difficulty removing the milk coating, while another had only discovered it later.

We discovered that if you leave it for a while before cleaning… when you remove the bottom it's a… rubber thing… there it can become like a coating of formula…you weren't aware of that. (R15)

One mother found boiling difficult, but believed it was more hygienic than using the dishwasher. Some wished for a sterilizing machine. Mothers who had a formula machine found it cumbersome to clean all the parts. One mother preferred washing bottles herself to ensure adequate cleaning. Another found that cleaning every day and boiling every other day helped to keep them clean.

#### 3.3.4 Safe Feeds and Cleanliness Important, but Perceptions and Adaptations Differ

Mothers emphasized the importance of serving safe feeds and usually used a cleaned bottle for the formula. However, they expressed uncertainty and misconceptions about shelf life and quality, and concerns about accidentally making the baby sick. Safety measures included preparing feeds immediately before use, discarding leftovers, avoiding reheating, and careful measuring.

…then you were worried that you might accidently make her sick… that it was important that you didn't give her too old formula. (R9)

Despite believing it was unnecessary, some mothers pre‐boiled municipal water, washed bottles after each use, or sometimes boiled them for safety. One mother thought that boiled water in a vacuum‐sealed glass bottle prevented bacteria better than a thermos. Another boiled feeding equipment occasionally after reading that new equipment should be boiled before the first use. One mother who did not use a dish brush and kept formula for several hours stressed the importance of clean bottles to prevent bacteria and stomach problems. Having been told by a midwife that breastmilk could be kept for 4 h at room temperature, she followed this recommendation also for formula. One mother knew boiling was recommended after each use but did not follow this, while another cleaned only with hot water, without using dish soap or a brush, based on recommendations to boil utensils in just water.

It feels like it's the cleanest with just hot water… because you are supposed to boil them in hot water, I think. (R8)

One mother prioritized simplifying the cleaning process.

…to use as little dish soap as possible… so that he doesn't ingest it and get sick… I believe that it shouldn't be too sterile. It says you should really boil… and clean properly… but I think … the bacteria in our home, our water… those are things he has to cope with… (R1)

### 3.4 The Centrality of Guidance and Support

#### 3.4.1 Absent or Insufficient Formal Information

Almost all mothers reported not receiving any information from healthcare professionals regarding the safe handling of infant formula and associated health risks. Several noted that the information was insufficient; some had to ask for advice themselves, while others found the guidance adequate.

…I wouldn't say I've gotten much advice. Perhaps I haven't asked much either… It's been more like you get a brochure with millilitres and stuff… Before that, I didn't get anything. (R2)

For those who did receive information, it was mainly provided after the baby was born, most often after formula feeding had already started. The focus was on formula being a good option, ensuring the correct mixing ratios, feeding temperature, and the importance of cleaning. Other topics included the risk of constipation and abdominal pain from too much powder, of too little energy intake from too little powder, and general recommendations on amounts and increases. Some mothers had just been told that they needed to start formula feeding for various reasons.

…maybe at the neonatal unit then… when everything was so new with the first baby… then they said it [formula] should be lukewarm… but nothing else… Nothing this time, but now we knew. (R4)

#### 3.4.2 Guided Through Own Choices and Informal Sources

Mothers described themselves as the primary decision‐makers regarding the preparation and storage of infant formula and cleaning methods. Almost all relied on one or more informal sources for guidance, mainly concerning handling but also associated health risks. Informal sources included instructions on the formula packaging, relatives, friends, and others with children. Additionally, various websites such as the 1177 (Sweden's national healthcare guide), the Swedish Food Agency, Facebook groups, pregnancy apps, child forums, and manufacturers' websites were mentioned. The majority found the instructions on the packaging helpful. However, one mother expressed difficulty understanding them and had to seek additional guidance.

…when I read on the jar, I don't understand anything… My sister also helped… but I didn't trust her, so I asked the midwife… Yes, the instructions are very difficult, I think. (R10)

Mothers highlighted the need to actively search for information, and the majority had googled different sources on the topic. Several found these sources useful. Some mentioned it was easy to google but emphasized the need to evaluate and be source critical.

It's more that you get to test yourself and read up on it… it's been a bit like a jungle. (R3)

#### 3.4.3 The Importance of Guidance

Most mothers expressed a desire for increased information, guidance, and support from healthcare professionals regarding the handling of infant formula. They wished they had been better prepared and emphasized the need for comprehensive verbal and written information. Additionally, they highlighted the importance of follow‐up routines from healthcare services to ensure proper management of infant formula.

I would have liked to have had this information directly at the hospital… it would have been nice to get an information sheet ‘this is how to mix, how to clean’… because when you get home… everything is upside down… And also later that you get a follow‐up… Because sometimes you can mix it incorrectly and then they get a stomach ache… because you didn't clean the bottles properly… (R3)

#### 3.4.4 A Taboo Subject or a Good Option? The Desire for Acceptance and Inclusion

Healthcare professionals were described as both supportive and informative about infant formula as a good alternative or complement to breastfeeding. However, some mothers perceived them as negative or questioning, making it difficult to seek advice. Others felt that the subject was not addressed, neither in healthcare nor in society. One mother found her social network more accepting and less prejudiced.

…I don't ask so much because it feels a bit taboo… It's not something you show… ask too much about, especially not in healthcare… With friends and family and things like that, it feels more open and less prejudiced… (R1)

Limited information led to feelings of guilt, and a lack of understanding from healthcare professionals caused feelings of shame. Mothers emphasized the importance of acceptance and support around infant formula, both from healthcare providers and society. They highlighted the need for a nonjudgemental attitude, a desire to remove stigma, and increased understanding and openness, instead of treating it as a taboo subject.

It somehow brings even more guilt that there is no information because then it's like…it's so wrong to give formula. (R7)

### 3.5 Aspects That Also Matter

#### 3.5.1 The Importance of Feeling Secure and Free

The availability of infant formula was highlighted as a great source of security as a single parent, as well as a source of gratitude for someone else. Mothers saw formula feeding as a way to share feeding responsibilities with their partner, receive help from others, and have more freedom and personal time.

…there are many advantages to being able to give formula too. I think then the father can feed too.…as a freedom not to be locked into…breastfeeding. (R5)

#### 3.5.2 Suitability and Preferences Matter, but Cost Can Be a Barrier

Multiple factors influenced mothers' decisions regarding products, equipment, and handling. Several mothers found infant formula expensive and uneconomical due to having to discard leftovers. One mother wished she did not have to use formula because of the cost. Most had chosen PIF over ready‐to‐feed formula because it was cheaper. One mother, who only owned two bottles, expressed a desire to be able to borrow bottles and try them out instead of buying several unnecessarily. Another mother, who had a formula machine, emphasized the unfairness that not everyone could afford one.

…that basic needs should not be a class issue… formula is expensive… and so is this machine. I understand that not everyone can spend [SEK] 3000 on such a machine … at the same time, it's … something that makes things much easier. (R7)

One mother, who only used ready‐to‐feed formula, had chosen it because it was quick and easy to prepare. Other factors influencing formula choices included ease of use, no need for refrigeration, pack size, suitability for formula machines, the child's needs and trust in the brand. Mothers believed that other parents' handling of infant formula were influenced by personal background, values, social influences, sources of information and practical considerations.

Many are probably influenced by the information… they have and receive, but perhaps also by convenience. (R6)

## 4 Discussion

This study reveals varying degrees of safe preparation, storage, and cleaning methods. Although feasible, mothers found these procedures challenging. Stress, impractical packaging, and negative experiences with cleaning and hygiene were highlighted. Additionally, mothers reported not receiving sufficient information from healthcare providers regarding safe handling and associated health risks. Relying mainly on informal sources, they expressed a strong desire for increased professional guidance and support. Limited information and support from healthcare services led to feelings of exclusion, shame, and guilt, with a call for greater inclusion. Positive feelings of security and freedom, but also cost‐related constraints, influenced use and handling.

According to mothers' descriptions, neither the Swedish guidelines (1177 2022) nor the WHO recommendations (WHO 2007) for the safe handling of infant formula were fully followed. Swedish healthcare guidelines do not explicitly state that water should be boiled before preparation, only that the manufacturer's instructions should be followed (1177 2022; The National Handbook for Child Health Services 2022). Given the different preparation practices, such as using tap water directly, there is a need for clarification. Although not common, incorrect preparation methods were reported, including pre‐measuring powder into the bottle before adding water, adding cream, using extra powder or water, prolonged storage and reheating. The use of microwaves seemed to be more common. However, these practices are discouraged (WHO 2007).

The route of bacterial transmission due to unhygienic practices is known (Cho et al. 2019). The results indicate a greater problem with lack of hygiene and cleaning routines among mothers, similar to findings in the United Kingdom (Grant et al. 2024). Practices such as not mixing PIF with water at a temperature of at least 70°C, poor hand hygiene, transference of powder, customized storage solutions, and various simplified cleaning methods increase the risks of bacterial contamination and unsafe feeds. Unlike WHO (WHO 2007) and other international guidelines (CDC 2024; Health Canada 2022; Health Norway 2021; National Health and Medical Research Council 2012; National Health Service (NHS) 2023), Swedish national healthcare guidelines do not consider sterilization of feeding utensils necessary (1177 2022; The National Handbook for Child Health Services 2022). Results indicate that this recommendation needs to be reevaluated more in line with WHO guidelines. Additionally, mothers emphasized the importance of fresh, safe feeds and cleanliness, but they also expressed conflicting perceptions and adaptations. Extra safety measures were taken alongside unsafe practices. This suggests a lack of knowledge and understanding concerning risks of bacterial contamination, as previously reported by parents (Calamusa et al. 2009; Grant et al. 2024; Labiner‐Wolfe et al. 2008).

The findings of limited information, as well as mothers' desire for more guidance and support from healthcare, align with previous research (Appleton et al. 2018; Hvatum and Glavin 2017; Lagan et al. 2014). This also applies to delays in receiving information about formula (Appleton et al. 2018, 2020), and the reliance on informal sources for guidance (Appleton et al. 2020; Fallon et al. 2017). It has been emphasized that instructions on formula packaging should match the health literacy skills of parents and other caregivers. However, research indicates that these instructions are often too advanced (Wallace et al. 2016). This issue is particularly important to consider, as it was also highlighted in the present study. Furthermore, several mothers perceived formula handling as challenging, navigating stress, impracticalities, and negative experiences. Similar concerns were reported among formula‐feeding mothers in Norway who felt unprepared and feared mixing unsafe formula (Hvatum and Glavin 2017). To ensure safe handling and promote manageability where formula is necessary, the findings suggest the need for timely, improved information and support, as well as the need for ongoing follow‐up.

Nurses in Australia working with infant feeding report incomplete or insufficient formal education on formula feeding. With mainly practical knowledge and limited access to workplace policies, their ability to provide relevant information is affected (Kotowski et al. 2022). This may apply to healthcare professionals and care units in Sweden, potentially explaining the limited information provided. Australian nurses also report difficulties interpreting infant feeding policies and providing accurate formula feeding information without marketing influences (Kotowski et al. 2022). The mixed experiences and emotions among the mothers due to varying information and support, as described previously (Appleton et al. 2018; Fahlquist 2014; Hvatum and Glavin 2017), may reflect similar difficulties with infant feeding policies among healthcare professionals in Sweden.

Parental characteristics are not consistently reliable predictors of improper handling (Carletti and Cattaneo 2008; Labiner‐Wolfe et al. 2008). Consistent with previous research (Appleton et al. 2022), mothers highlighted several factors they considered important when using and handling formula. Among these, costs seemed to be a particularly negative influence on decision‐making regarding products, equipment and handling. The WHO recommends using ready‐to‐feed formula (which is sterilized during production) for infants at the greatest risk of infection, including those under 2 months of age (WHO 2007). However, awareness of this recommendation is unclear. Most mothers had chosen PIF for economic reasons. Convenience was also mentioned as a factor influencing handling practices, which may help explain why some mothers did not adhere to guidelines despite being aware of the risks. The results contribute to an increased understanding of the issues and factors parents must deal with when formula feeding.

### 4.1 Methodological Considerations

Concepts for judging trustworthiness of the study, including credibility, dependability, confirmability, and transferability were considered (Graneheim et al. 2017; Graneheim and Lundman 2004). Study participants had diverse experiences of infant formula use, variations in personal characteristics including socio‐economic diversity, and infant's age, thus increasing the study's credibility. Individual interviews allowed for spontaneous, personal, and rich descriptions, covering significant variations in content and multiplicity. A limitation was that no co‐parents, other close relatives, or friends participated, which might have provided additional important insights. Additionally, recruitment through healthcare professionals responsible for infant formula counseling may have influenced both participant selection and responses, thereby introducing potential bias into study findings. However, the study aimed for understanding rather than generalization. To minimize the influence of the researcher during data collection, an interview guide was used, and to minimize the influence of pre‐understanding on the results, more than one researcher participated in the analysis process. The representative group of mothers further strengthens the transferability of the findings to similar settings.

## 5 Conclusion

This study highlights the need for improved information, guidance and support for parents on the safe handling of infant formula. The results indicate that this is a nonprioritized topic in the healthcare continuum. Thus, there is a need to review the quality of national and local guidelines in relation to the WHO guidelines on safe formula feeding and assess how they are implemented, as well as evaluate how well they are understood and followed at all levels of the healthcare system.

## Author Contributions

**Erika Andresen:** conceptualization (equal), data curation (lead), formal analysis (equal), investigation (equal), methodology (equal), visualization (lead), writing – original draft (lead), writing – review and editing (equal). **Paola Oras:** conceptualization (equal), formal analysis (equal), methodology (equal), supervision (equal), writing – review and editing (equal). **Gunilla Norrman:** conceptualization (equal), formal analysis (equal), methodology (equal), writing – review and editing (equal). **Mats Målqvist:** conceptualization (equal), formal analysis (equal), methodology (equal), supervision (equal), writing – review and editing (equal). **Eva‐Lotta Funkquist:** conceptualization (equal), formal analysis (equal), investigation (equal), methodology (equal), project administration (lead), resources (lead), supervision (equal), writing – review and editing (equal).

## Conflicts of Interest

The authors declare no conflicts of interest.

## Data Availability

The data that support the findings of this study are available from the corresponding author upon reasonable request.
